# Supplementary figures and images for: Week-long imaging of cell divisions in the Arabidopsis root meristem
Source: Plant Methods. 2019 Mar 25;15:30. doi: 10.1186/s13007-019-0417-9 (PMC6446972; doi:10.1186/s13007-019-0417-9)

Root Length  
(mm)

80  
60  
40  
20  
0

0

coverslip

2

4

6

0

plate

2

4

6

Time  
(days post transfer)

0

coverslip

2

4

6

0

plate

2

4

6

H2B  
WT

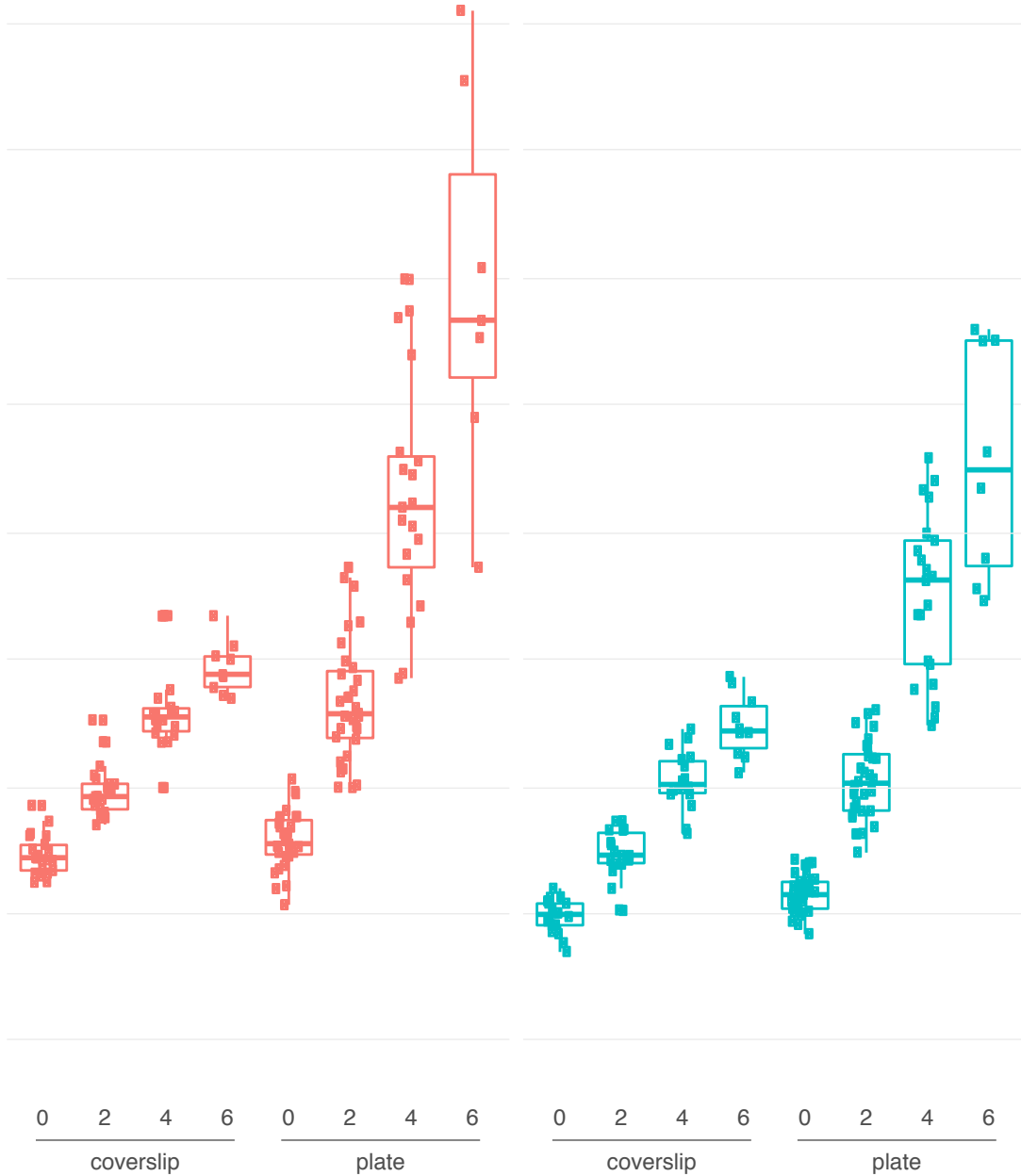

Supplement: Supplementary file 3 — Additional file 3. Boxplot with jitter, showing root growth on plates versus coverslip chamber (1% agar) grown under standard conditions. Both wild-type and pWOX5::GFP(ER)/35S::H2B-mRFP1 transgenic plants are plotted. In X axis, time (days post transfer) is shown for different conditions (coverslip vs. plate) and genotypes (H2B = H2B/WOX dual reporter line; WT = Col-0 wild type). Y axis shows length in millimeters. [file 13007_2019_417_MOESM3_ESM.pdf]

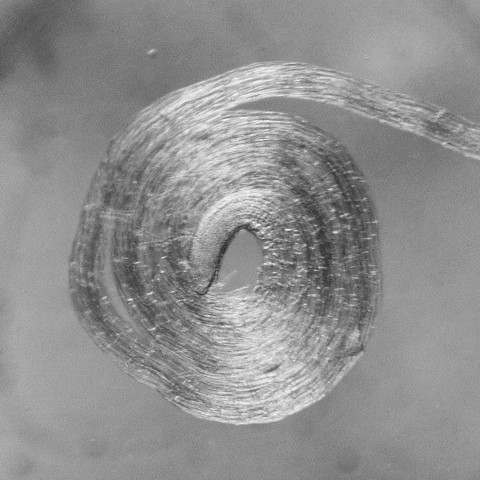

Supplement: Supplementary file 4 — Additional file 4. A root that had coiled in on itself having grown horizontally in the growth chamber described in Fig. 2 when fishing line is omitted from the setup. [file 13007_2019_417_MOESM4_ESM.jpg]

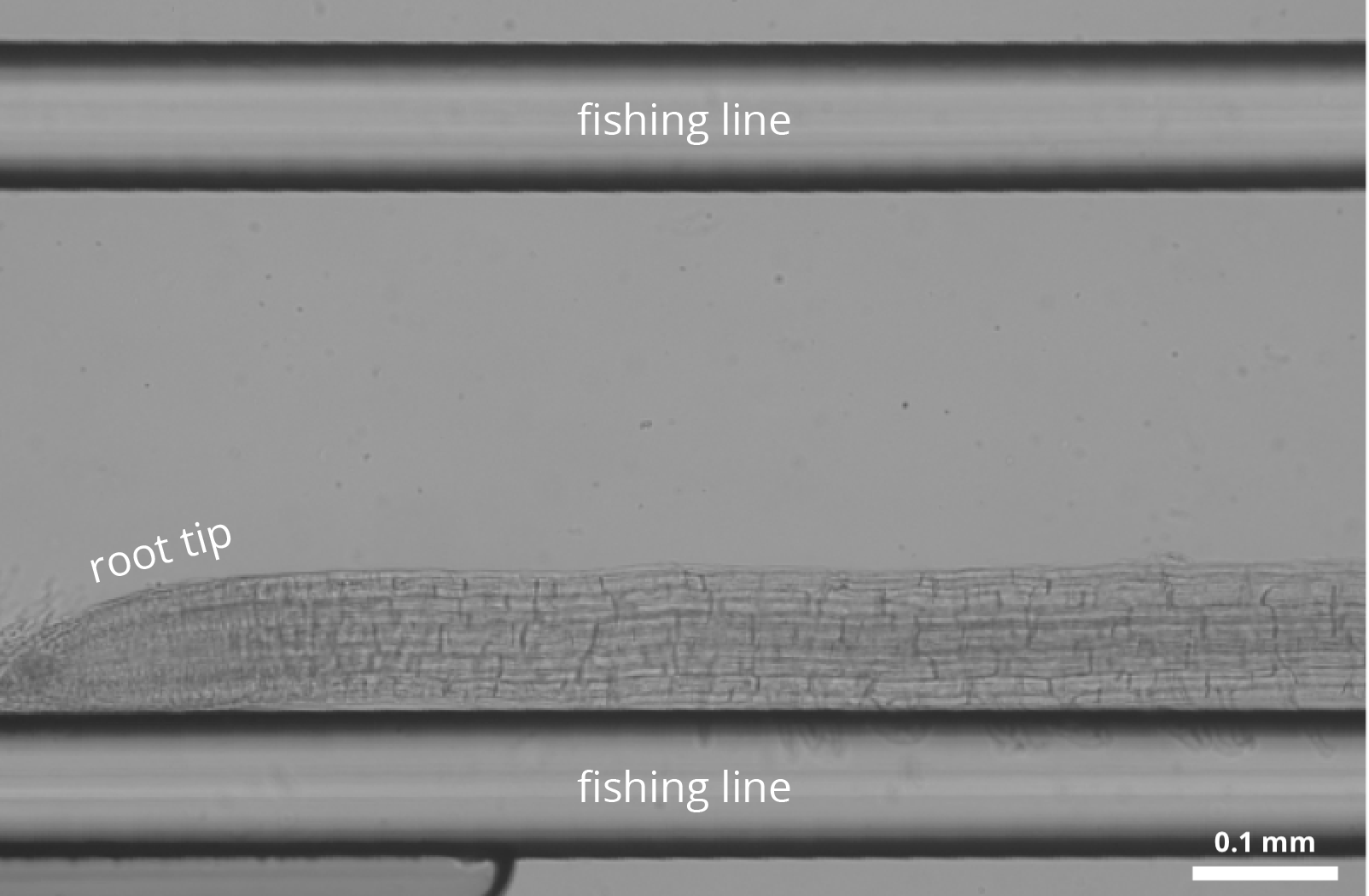

Supplement: Supplementary file 6 — Additional file 6. Closeup of a root showing growth alongside a fishing line “guide”, which greatly minimizes rotation and coiling. [file 13007_2019_417_MOESM6_ESM.png]

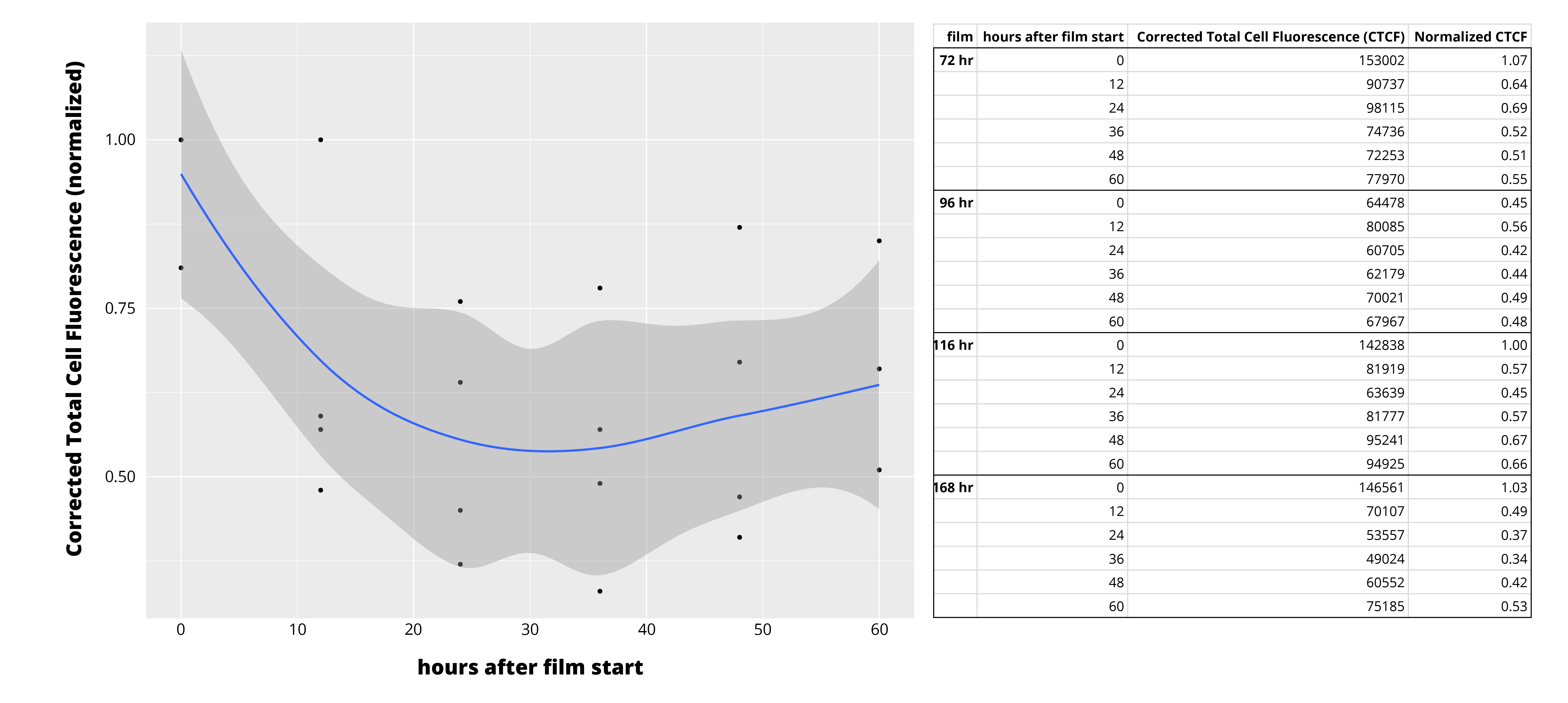

Supplement: Supplementary file 8 — Additional file 8. Plot of Corrected Total Cell Fluorescence over time for each of the four films over six time points. Table (right) contains the underlying data. Blue line represents loess fit. [file 13007_2019_417_MOESM8_ESM.png]

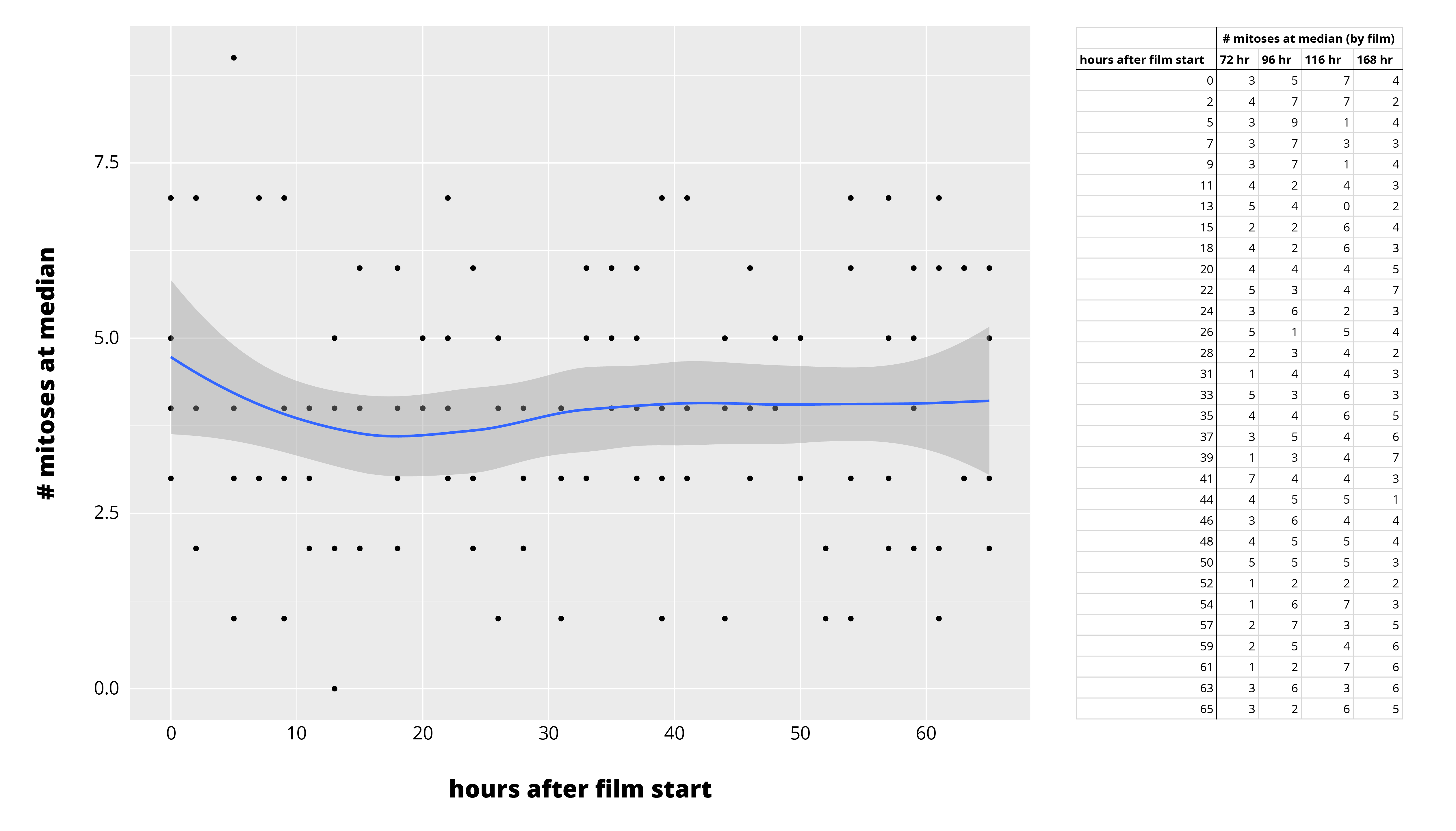

Supplement: Supplementary file 9 — Additional file 9. Plot showing numbers of mitotic cells observed at a median section over time for each of the four films. Table (right) contains the underlying data. Blue line represents loess fit. [file 13007_2019_417_MOESM9_ESM.png]

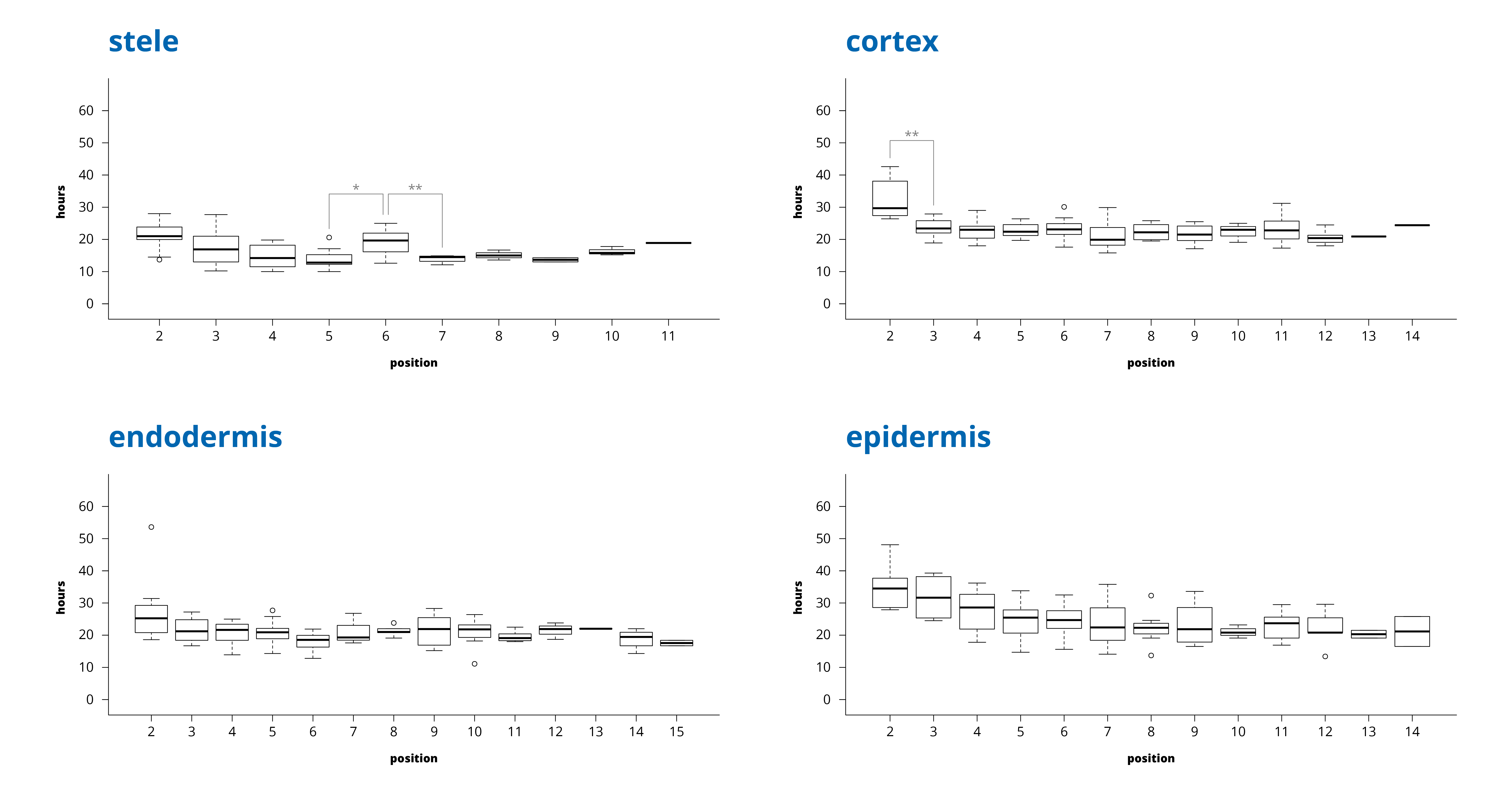

Supplement: Supplementary file 13 — Additional file 13. Boxplots showing each of the tissue-specific division patterns for the TA zone (positions 2–15). [file 13007_2019_417_MOESM13_ESM.png]

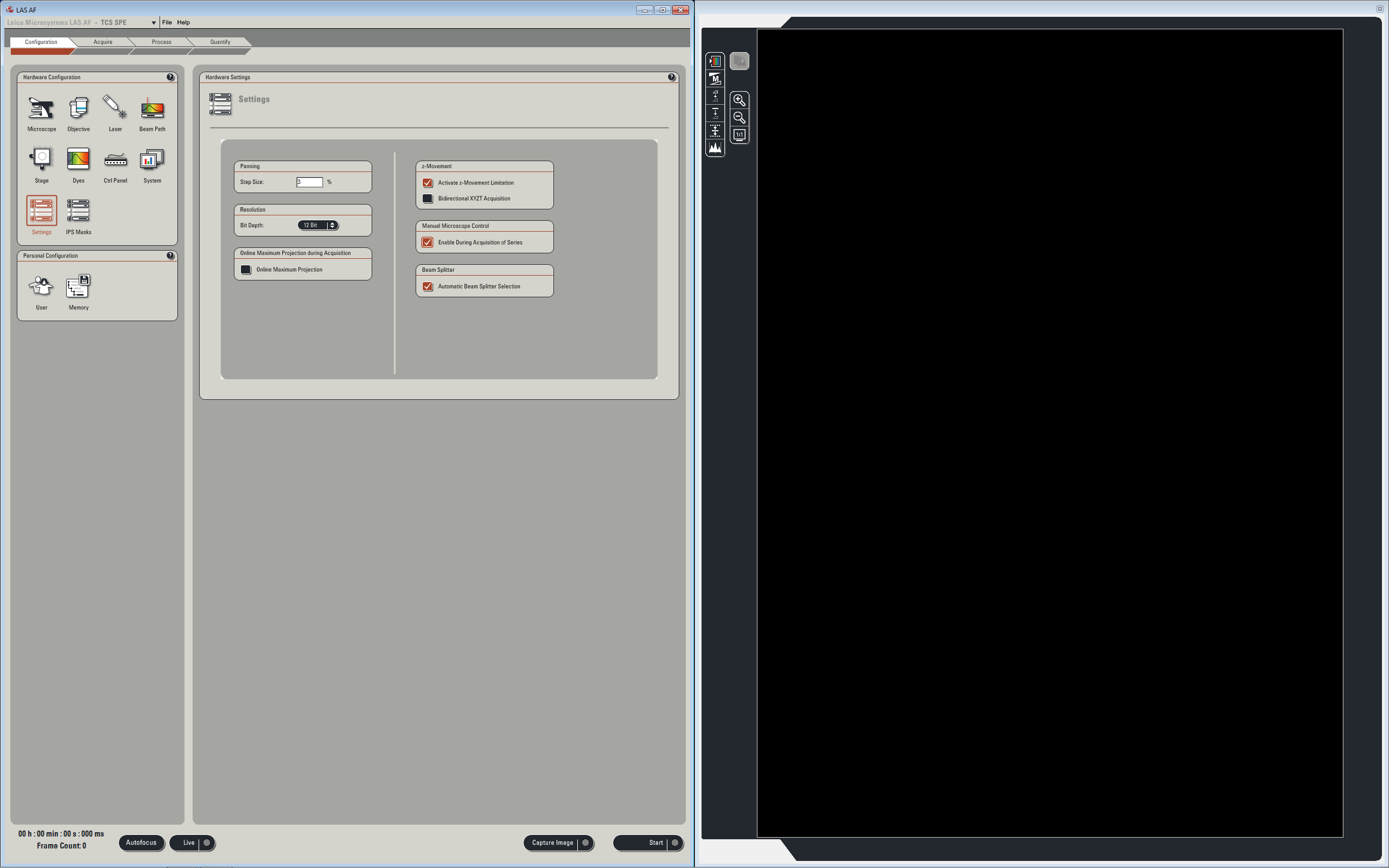

Supplement: Supplementary file 17 — Additional file 17. Screenshot A. Screenshots of MatrixScreener settings and steps required to set up automated tracking and drift correction, as described in Detailed Instructions for MatrixScreener Template file. [file 13007_2019_417_MOESM17_ESM.png]

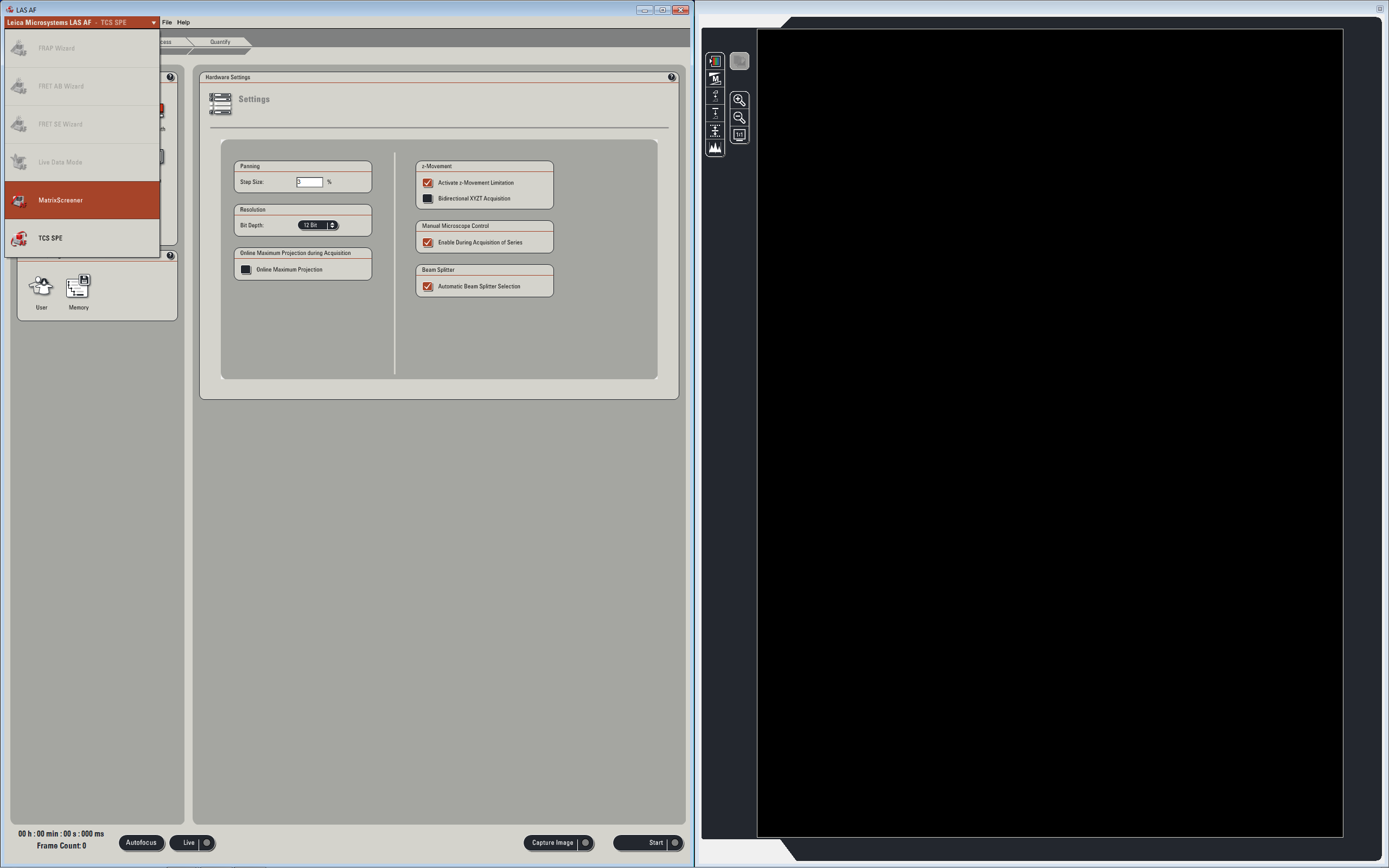

Supplement: Supplementary file 18 — Additional file 18. Screenshot B. Screenshots of MatrixScreener settings and steps required to set up automated tracking and drift correction, as described in Detailed Instructions for MatrixScreener Template file. [file 13007_2019_417_MOESM18_ESM.png]

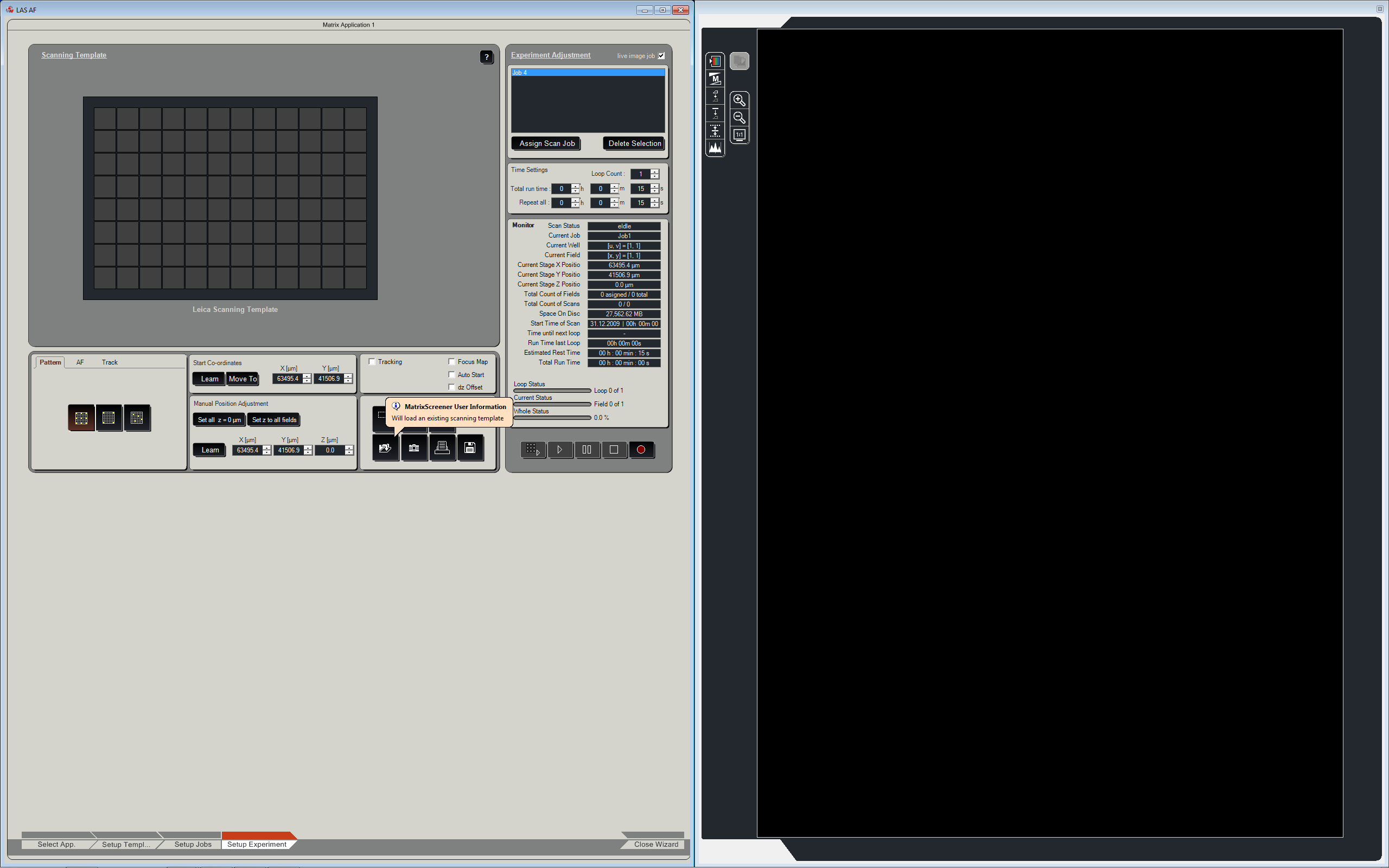

Supplement: Supplementary file 19 — Additional file 19. Screenshot C. Screenshots of MatrixScreener settings and steps required to set up automated tracking and drift correction, as described in Detailed Instructions for MatrixScreener Template File. [file 13007_2019_417_MOESM19_ESM.png]

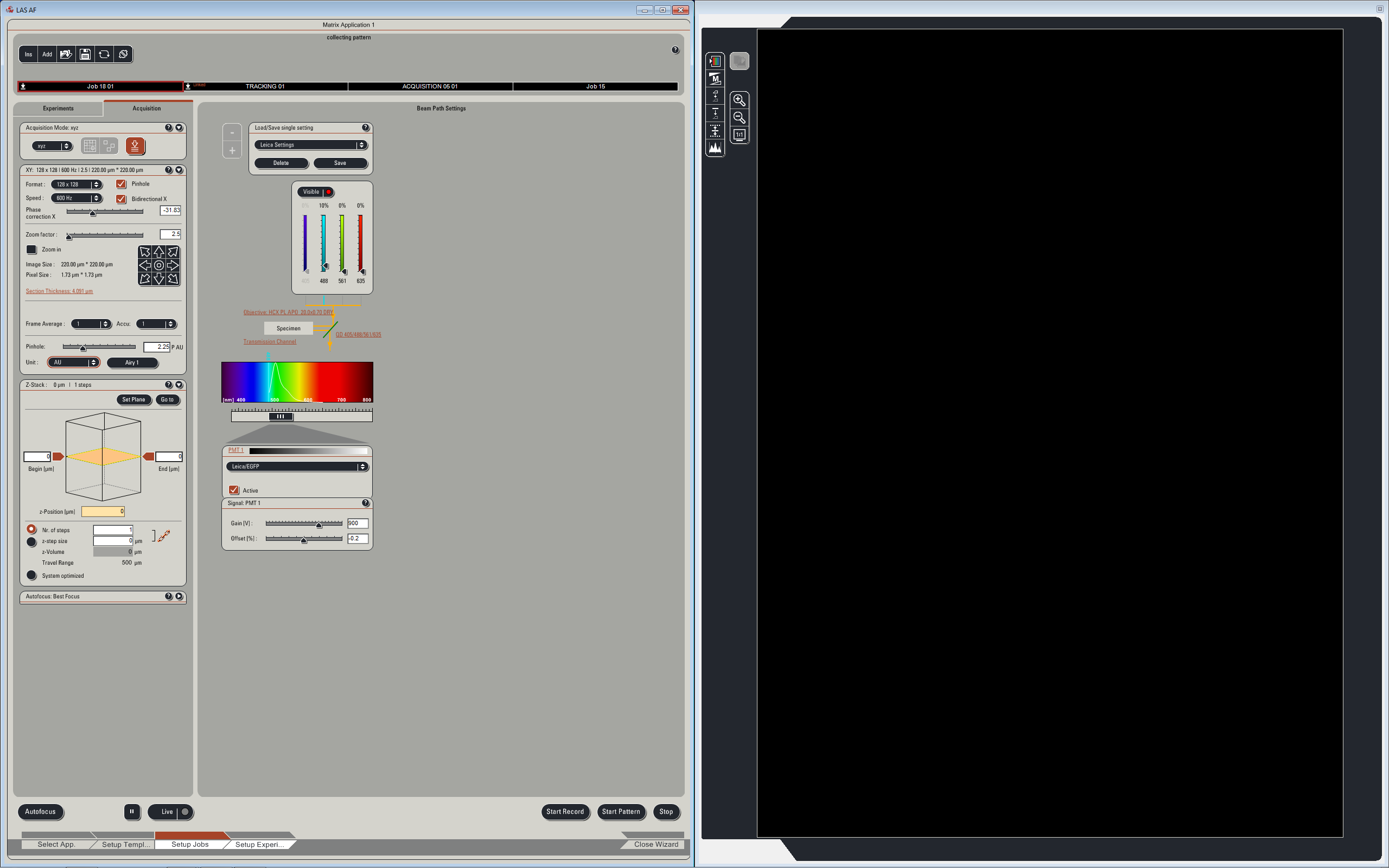

Supplement: Supplementary file 20 — Additional file 20. Screenshot D. Screenshots of MatrixScreener settings and steps required to set up automated tracking and drift correction, as described in Detailed Instructions for MatrixScreener Template File. [file 13007_2019_417_MOESM20_ESM.png]

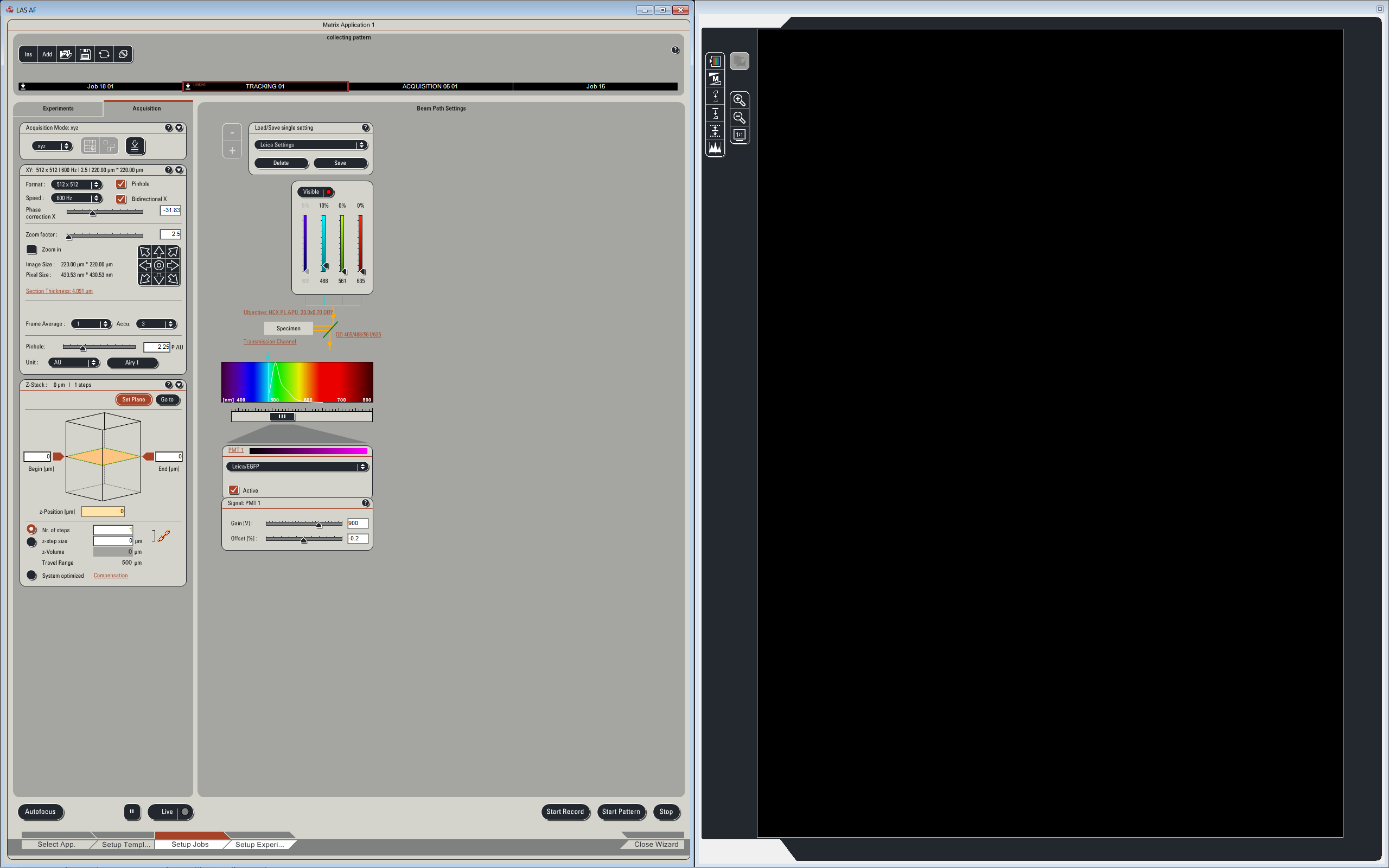

Supplement: Supplementary file 21 — Additional file 21. Screenshot E. Screenshots of MatrixScreener settings and steps required to set up automated tracking and drift correction, as described in Detailed Instructions for MatrixScreener Template File. [file 13007_2019_417_MOESM21_ESM.png]

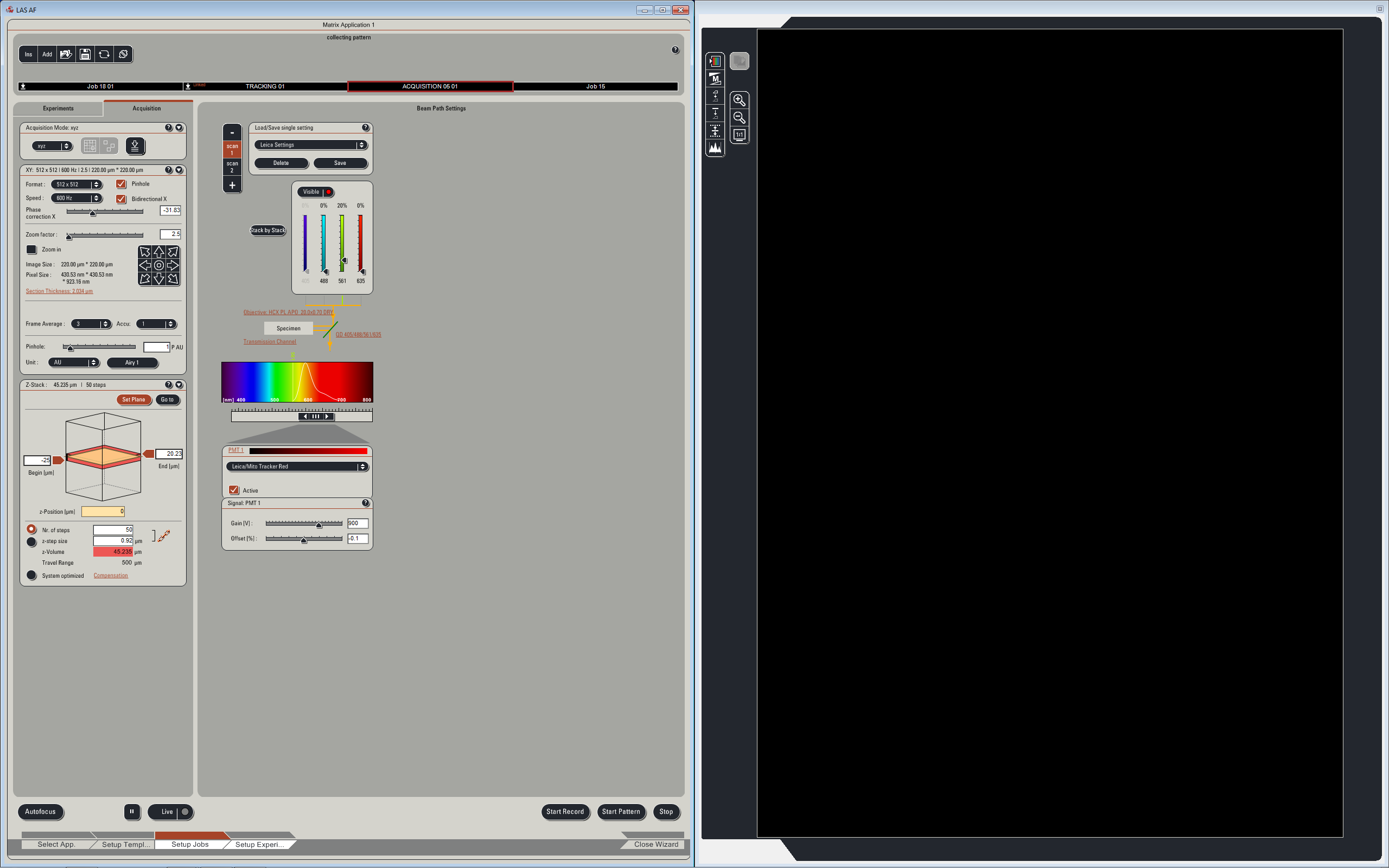

Supplement: Supplementary file 22 — Additional file 22. Screenshot F. Screenshots of MatrixScreener settings and steps required to set up automated tracking and drift correction, as described in Detailed Instructions for MatrixScreener Template File. [file 13007_2019_417_MOESM22_ESM.png]

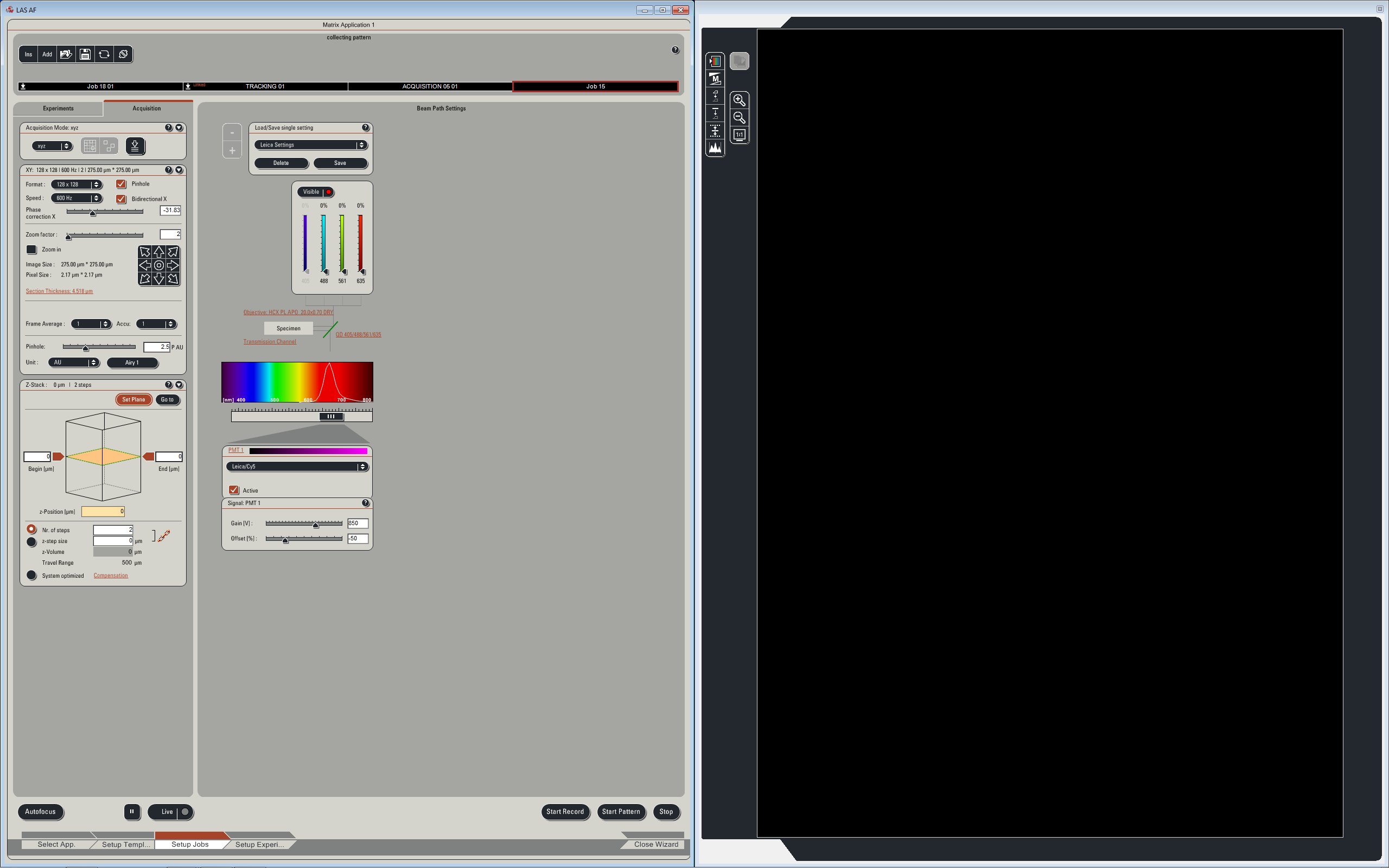

Supplement: Supplementary file 23 — Additional file 23. Screenshot G. Screenshots of MatrixScreener settings and steps required to set up automated tracking and drift correction, as described in Detailed Instructions for MatrixScreener Template File. [file 13007_2019_417_MOESM23_ESM.png]

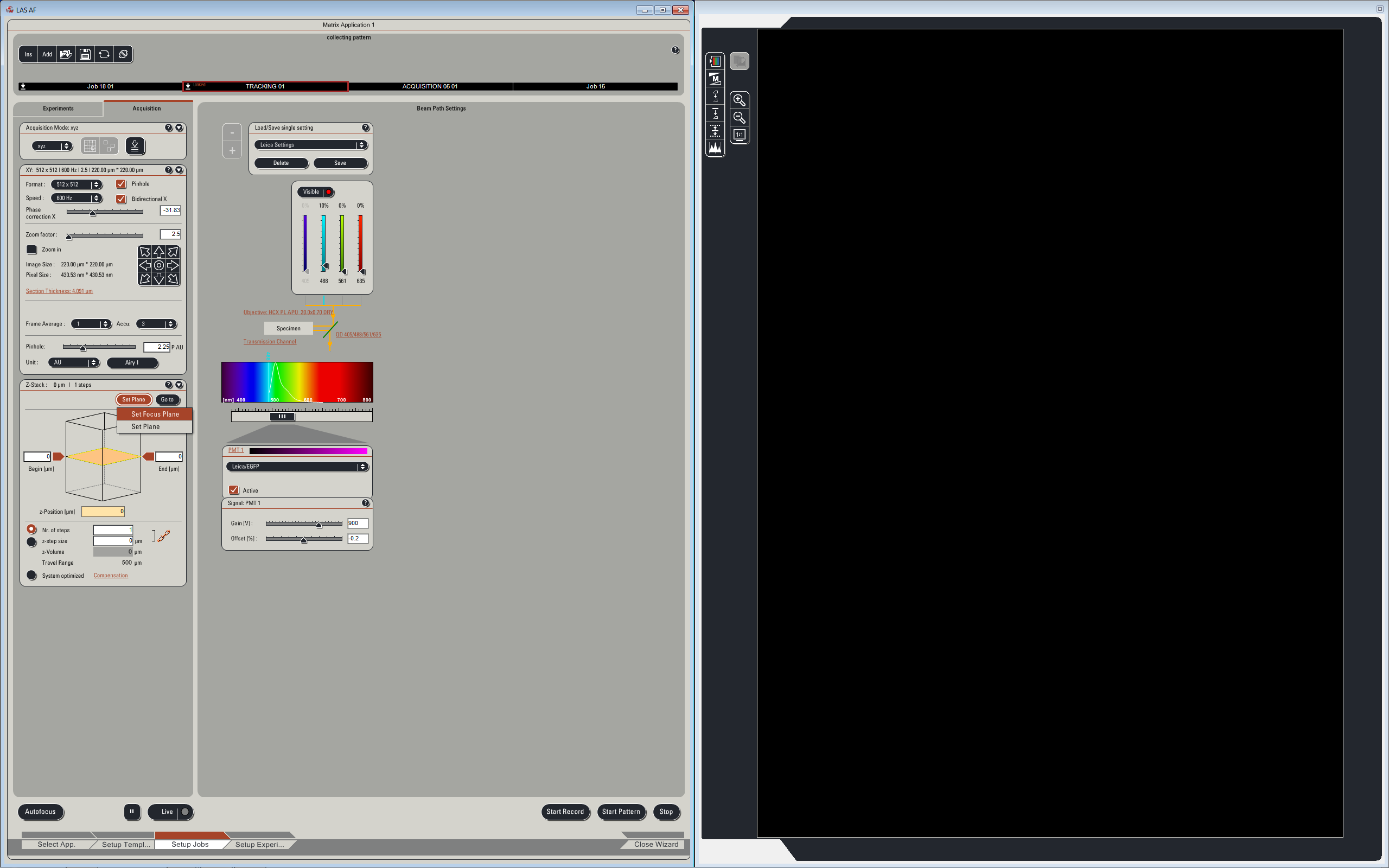

Supplement: Supplementary file 24 — Additional file 24. Screenshot H. Screenshots of MatrixScreener settings and steps required to set up automated tracking and drift correction, as described in Detailed Instructions for MatrixScreener Template File. [file 13007_2019_417_MOESM24_ESM.png]

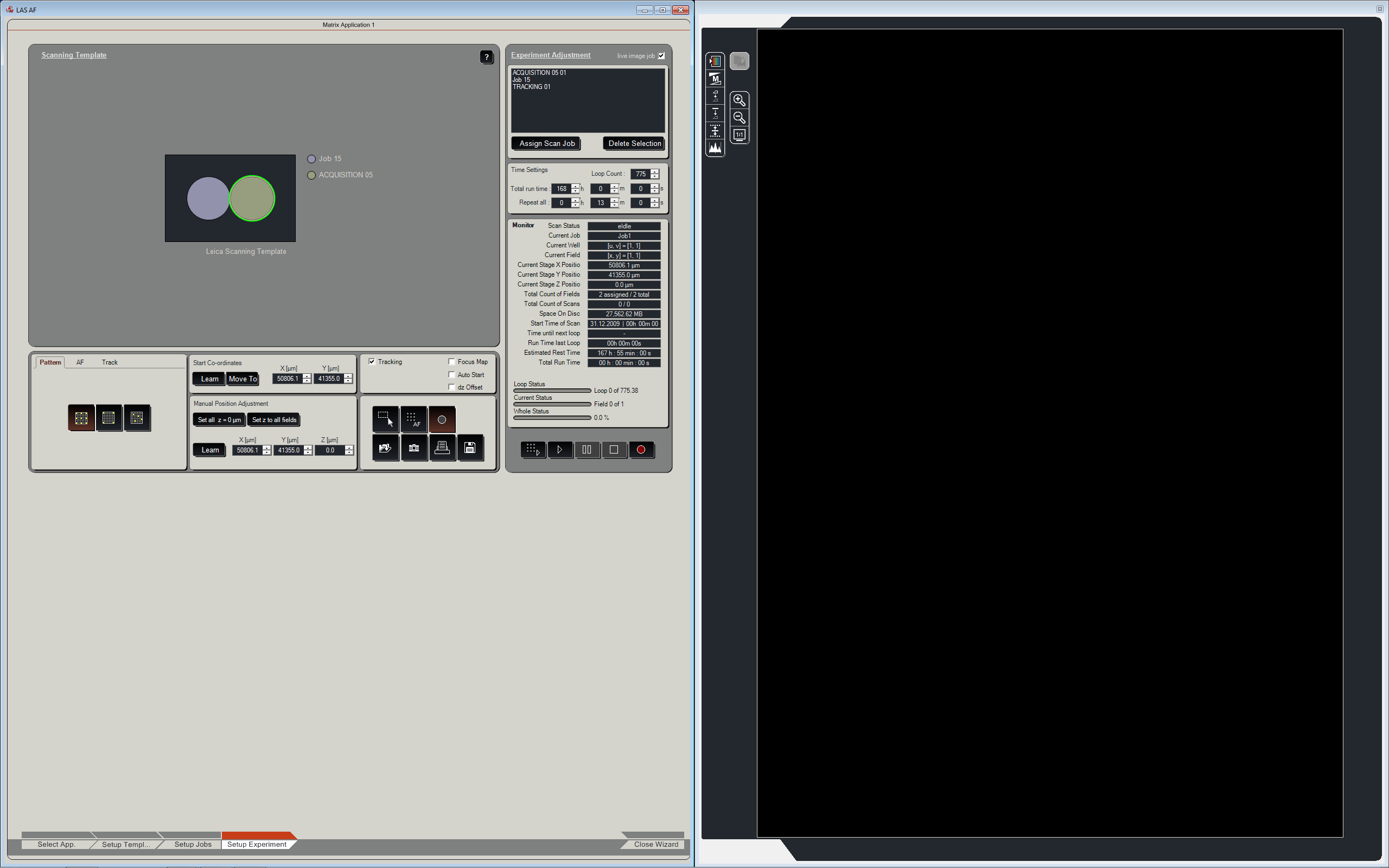

Supplement: Supplementary file 25 — Additional file 25. Screenshot I. Screenshots of MatrixScreener settings and steps required to set up automated tracking and drift correction, as described in Detailed Instructions for MatrixScreener Template File. [file 13007_2019_417_MOESM25_ESM.png]
