# Supplementary material for: Week-long imaging of cell divisions in the Arabidopsis root meristem
Source: Plant Methods. 2019 Mar 25;15:30. doi: 10.1186/s13007-019-0417-9 (PMC6446972; doi:10.1186/s13007-019-0417-9)
Supplement: Supplementary file 11 — Additional file 11. Results from two-way ANOVA and Tukey Test for all tissues (A), initials + TA zone (B), and TA zone alone (C). [file 13007_2019_417_MOESM11_ESM.pdf]

A: Positions -2 to -1, and 1 to 15

two-way ANOVA

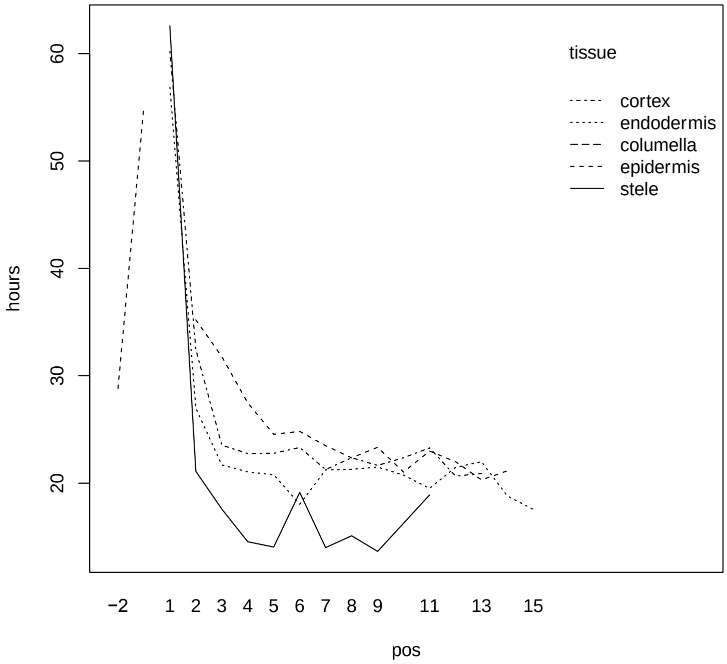

> > > Figure legend: < < < # #

The figure represents interaction plot for hours (y-axis). The interaction between pos (x-axis) and tissue (different lines) w
as quantified using two-way ANOVA.

Analysis of Variance Table

Response: pheno

	Df	Sum Sq	Mean Sq	F value	Pr(>F)	
iv1	16	38619	2413.66	57.8164	< 2.2e-16	***
iv2	4	3143	785.76	18.8220	5.945e-14	***
iv1:iv2	35	1087	31.04	0.7436	0.8558	
Residuals	330	13777	41.75			

Signif. codes: 0 ‘***’ 0.001 ‘**’ 0.01 ‘*’ 0.05 ‘.’ 0.1 ‘ ’ 1

iv1 corresponds to position and iv2 corresponds to tissue, with iv1:iv2 reflecting their interaction.

B: Positions 1 to 15

Testing significant differences

ANOVA

The p-value of the ANOVA test between different pos is 0.

SIGNIFICANT difference in means

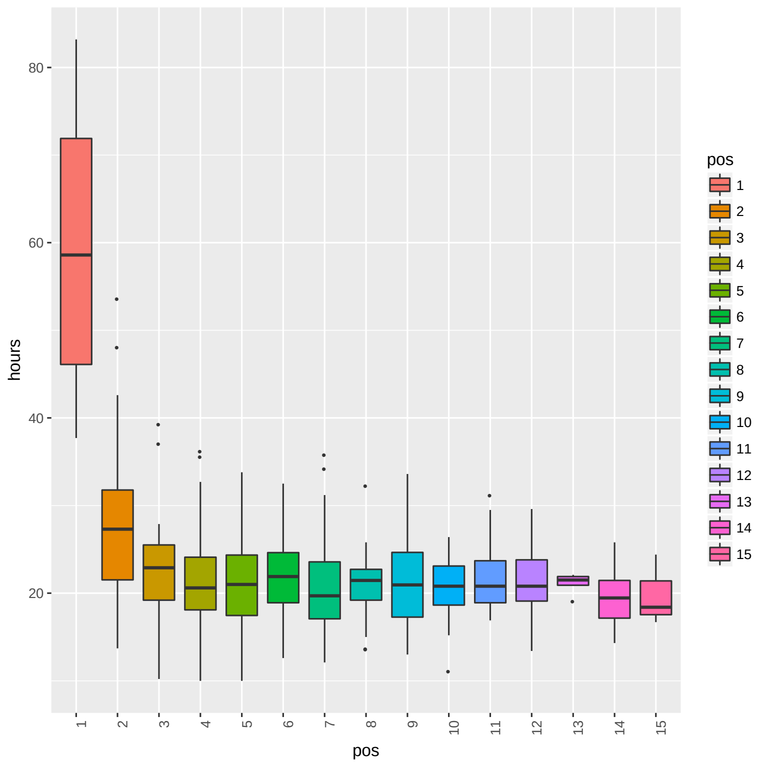

> > > Figure legend: < < < # #

The plot represents the boxplot of hours by pos . The data used is raw data .

pos   hours Significant groups based on Tukey's pairwise comparison

1	59.38235	a
2	28.30250	b
3	22.27500	c
11	21.79600	c
6	21.76053	c
9	21.50769	c
4	21.38605	c
12	21.36154	c
7	21.33000	c
13	21.10000	c
8	21.07727	c
5	20.65385	c
10	20.34211	c
15	19.83333	c
14	19.58333	c

C: Positions 2 to 15

Testing significant differences

ANOVA

The p-value of the ANOVA test between different tissue is 0.

SIGNIFICANT difference in means

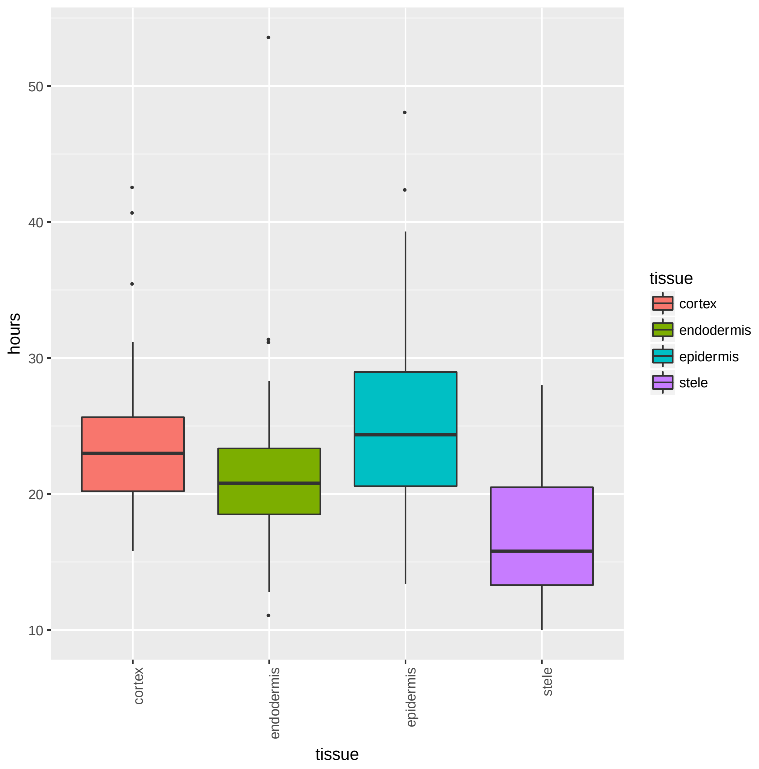

> > > Figure legend: < < < # #

The plot represents the boxplot of hours by tissue . The data used is raw data .

tissue	hours	Significant groups based on Tukey's pairwise comparison
epidermis	25.25000	a
cortex	23.45862	a
endodermis	21.30090	b
stele	16.96949	c
